# Apple Pomace as a Promising By-Product with High Antioxidant Potential in the Prevention of Aging Processes

**DOI:** 10.3390/foods15071174

**Published:** 2026-03-31

**Authors:** Kamil Wysocki, Maima Matin, Magdalena Koszarska, Cyprian Tomasik, Bogumiła Zima-Kulisiewicz, Nina Strzałkowska

**Affiliations:** 1 Department of Biotechnology and Nutrigenomics, Institute of Genetics and Animal Biotechnology, Polish Academy of Sciences, 05-552 Magdalenka, Poland; 2 Independent Researcher, 39-209 Zasów, Poland

**Keywords:** apple pomace, anti-aging, oxidative stress, phenolic compounds, applications

## Abstract

Aging is closely linked to oxidative stress and inflammation. This review provides a critical overview of the antioxidant compounds present in apple pomace and explores how they may mitigate age-related oxidative damage and inflammatory responses. We focus on the nutritional profile of apple pomace including its macro- and micronutrients, with particular focus on polyphenols, such as procyanidin tannins, quercetin glycosides (rutin, quercetin-3-glucoside), phloridzin, dietary fiber, vitamins, and lipids alongside current techniques for isolating its bioactive components. Special attention is given to biological pathways through which these compounds influence aging: redox regulation via Nrf2, inflammatory modulation via NF-κB, and metabolic regulation via AMPK, SIRT1 and PI3K/Akt/mTOR. Evidence from in vitro cellular models (HepG2, CCD-986Sk fibroblasts), in vivo rodent studies and limited human pilot trials is summarized, as well as existing and emerging applications of apple pomace in functional foods, cosmeceuticals, and other sectors. Finally, we discuss the challenges and future opportunities in harnessing this by-product of the food industry. Although clinical data remain limited, preclinical findings support the repurposing of apple pomace as a sustainable functional ingredient contributing to healthier aging and circular economy goals. Future long-term randomized controlled trials are necessary to confirm efficacy in humans.

## 1. Introduction

The aging process does not happen without cause. It consists of numerous reactions, including oxidative stress and chronic inflammation (often referred to as “age-related inflammation”). Both of these factors contribute to cell damage and age-related diseases [1]. An excess of reactive oxygen species (ROS) and pro-inflammatory mediators that accumulate over time can damage tissues. This ultimately leads to degenerative changes. Aging is not driven solely by oxidative stress and chronic inflammation. Other well-established hallmarks such as genomic instability, telomere attrition, epigenetic alterations, and deregulated nutrient sensing also contribute significantly, as has been extensively reviewed elsewhere [2,3].

Consequently, there is growing interest in dietary antioxidants and natural anti-inflammatory agents as strategies to mitigate oxidative damage and support healthy aging. Plant polyphenols in particular have attracted attention for their free-radical scavenging and anti-inflammatory effects that may counteract key mechanisms of aging [4,5,6].

Apple pomace, the solid residue from apple juice and cider production, has emerged as a promising, sustainable source of such bioactive compounds. The global apple processing industry generates millions of tons of pomace annually, creating disposal costs and environmental concerns [7]. Rather than treating it as waste, apple pomace can be “upcycled” as a valuable reservoir of nutrients and phytochemicals. Notably, it is rich in dietary fiber, pectin, polar lipids, vitamins and polyphenols that exhibit potent antioxidant and anti-inflammatory activities [8,9,10,11,12]. These phytochemicals are well-known to neutralize ROS and modulate key signaling pathways, including activation of nuclear factor erythroid 2-related factor 2 (Nrf2) driven antioxidant response, inhibition of nuclear factor kappa-light-chain-enhancer of activated B cells (NF-κB) nuclear translocation, stimulation of AMP-activated protein kinase (AMPK) and Sirtuin 1 (SIRT1) and regulation of Phosphoinositide 3-kinases/Protein kinase B/mammalian target of rapamycin (PI3K/Akt/mTOR), thereby influencing mitochondrial function, autophagy and cellular senescence [12]. By interrupting free-radical chain reactions, apple pomace polyphenols can potentially slow processes associated with cellular aging [13]. The evidence presented derives primarily from in vitro and in vivo preclinical models, with emerging support from small human pilot studies.

Collectively, the bioactive profile of apple pomace suggests that this low-cost by-product could be repurposed as a functional ingredient to combat oxidative stress and inflammation in populations, aligning with the circular economy and sustainability goals [10]. Realizing this potential requires efficient extraction and utilization of pomace bioactive compounds. A range of conventional and “green” extraction techniques have been explored to recover polyphenols and other antioxidants from apple pomace [14,15].

The recovered extracts and powders can be reused. This has been the case for several years. They are used in various industries. For example, polyphenol concentrates from apple pomace are added to functional foods (bread, dairy products, etc.). This use not only increases the antioxidant content but also the nutritional value. This material was already tested several years ago as a source of dietary fiber in product enrichment [16]. Apple pomace derivatives have also found application in skin care products due to their protective and anti-wrinkle properties.

Polyphenol-rich extracts from pomace have been incorporated into anti-aging and skin-brightening products as natural alternatives to synthetic ingredients [17]. Similarly, pomace components have pharmaceutical uses such as, pomace-derived pectin is utilized in controlled-release drug formulations, and polyphenol fractions are being investigated as nutraceutical supplements [18]. This convergence of sustainability and functionality makes apple pomace an attractive subject for research and development in food, cosmetic, and pharma applications.

In this review, we critically investigate the antioxidant constituents of apple pomace and their mechanisms in counteracting aging-related oxidative stress and inflammation. We discuss the composition of apple pomace (macro- and micro-nutrients, with emphasis on polyphenols), current methods for extracting its bioactive compounds, and the biological activities by which these compounds may influence aging processes. We also review the evidence from studies, highlight current and emerging uses of apple pomace in functional foods, cosmeceuticals, and other industries, and outline the challenges and future prospects in valorizing this agro-food waste. We aim to demonstrate how apple pomace can be transformed from an industrial waste into a value-added product with high antioxidant potential to help counteract aging processes, benefiting both human health and environmental sustainability.

## 2. Apple Pomace: Composition and Its Bioactive Compounds

Apple pomace, the residual mash of skins, pulp, core, and seeds left after juicing, is a repository of bioactive nutrients with remarkable antioxidant potential. Comprising ~95% apple peel and flesh (and only ~2–4% seeds and 1% stems), pomace retains the bulk of the apple’s phytochemicals that do not transfer into juice. In fact, the majority of apple polyphenols (on the order of 80–99%) remain in the pomace byproduct [16]. This phytochemical richness makes pomace a concentrated source of compounds hypothesized to counteract aging processes via antioxidative and other mechanisms. Consistent with this, Skinner et al. (2018) noted that apple pomace is a rich source of health-benefitting nutrients, including minerals, dietary fiber, antioxidants, and ursolic acid, supporting its potential as a functional food ingredient [19]. The nutrient profile of pomace is dominated by carbohydrates (largely insoluble dietary fiber) with smaller fractions of protein, lipids, vitamins, and minerals [16,20]. We assume that polyphenolic antioxidant compounds and their accompanying co-nutrients provide the anti-aging protection.

### 2.1. Polyphenols and Antioxidant Phytochemicals

Polyphenols are the important bioactive compounds in apple pomace, accounting for a significant portion of its composition and antioxidant capacity. Pomace is especially enriched in flavonoids and related phenolics that scavenge free radicals and modulate redox-sensitive signaling pathways. Key constituents include flavan-3-ols (catechin, epicatechin and their oligomers procyanidins), flavonols (primarily quercetin conjugates), dihydrochalcones (phloridzin and its aglycone phloretin), and phenolic acids (especially chlorogenic acid, a caffeic acid derivative) [21,22]. The main polyphenolic compounds contained in apple pomace are summarized in Table 1.

Quantitatively, procyanidin tannins appear most abundant. A recent metabolomic analysis found polymeric procyanidins to be the dominant phenolic class in pomace extracts. These condensed tannins can constitute a large fraction of total polyphenols and were strongly correlated with in vitro antioxidant power FRAP assay [23]. Notably, quercetin glycosides (e.g., rutin, quercetin-3-glucoside) and phloridzin are prominent in apple skins and thus in pomace; one varietal analysis (Royal Delicious apples) detected phloridzin at ~487 µg/g and quercetin glycosides ~170–195 µg/g in dried pomace [24]. These compounds are potent antioxidants: quercetin’s polyhydroxylated flavonol structure confers strong radical-scavenging activity and anti-inflammatory effects (e.g., via Nrf2 and NF-κB pathway modulation), which have been linked to mitigation of age-related oxidative stress [25,26].

Phloridzin, an unique apple dihydrochalcone, likewise exhibits multifaceted bioactivity and can directly quench reactive oxygen species which has been shown to inhibit inflammatory signaling (e.g., IL-1β/NF-κB) in vitro [27]. Interestingly, phloridzin also acts as a natural SGLT inhibitor, thereby blunting glucose uptake. This antidiabetic mechanism may indirectly benefit aging by reducing glycemic spikes and glycation damage. Indeed, advanced glycation end-products (AGEs) are contributors to aging, and both phloridzin and phloretin can trap the reactive carbonyl methylglyoxal to prevent AGE formation [28]. Such structure–activity insights highlight that apple pomace polyphenols not only neutralize free radicals but also combat other deleterious chemistry (e.g., carbonyl stress) implicated in aging.

It is important to note potential conceptual tensions regarding polyphenol bioavailability. While pomace’s high polyphenol content drives strong in vitro antioxidant readings, some constituents (notably the polymeric procyanidins) are poorly absorbed in vivo. Bindon et al. (2023) cautioned that these large tannins “may not cross the intestinal cell layer,” meaning the impressive ferric-reducing antioxidant power of pomace extracts could overestimate direct bioactivity in the body [23].

However, this does not mean that we overestimated their importance. Unabsorbed polyphenols can have antioxidant and anti-inflammatory effects in the intestines [29]. They are also metabolized by the colonic microflora into smaller phenolic compounds that are digestible and beneficial to health. Overall, the effect appears to be very beneficial. The polyphenolic fiber matrix from apple pomace provides a prolonged release of antioxidants throughout the digestive tract, potentially protecting tissues and modulating signaling in a way that supports healthy aging. This hypothesis is supported by animal studies. Ravn-Haren et al. (2018) found that apple pomace improved gut health and antioxidant status in rats, even after removing the seeds (the main source of vitamin E) [30]. Such findings lead to the conclusion that the interaction between polyphenols and fiber in pomace may bring significant systemic benefits far beyond what would be predicted based on the absorption of a single compound.

**Table 1:** The main polyphenolic compounds contained in apple pomace and their effects in vivo and in vitro.

Compound	Research Model	Upregulation	Downregulation	References
Kaempferol	NIH3T3 cells, JB6 P+ cells, Female SKH-1 hairless mice, Dorsal skin from SKH-1 mice		RSK2, MSK1, skin carcinogenesis, phosphorylation of CREB and histone H3	[31]
Quercetin-3-Glucoside	HeLa cells	apoptosis, caspase-9/-3, BAX	cytotoxic effects, BCL-2	[32]
Quercetin	HepG2 cells	Nrf2	ROS, NF-κB, COX-2	[33]
Quercitrin	CCD-986Sk fibroblast cells		MMP-1	[34]
	In vitro assays: BSA-MGO Assay, BSA-Glucose Assay, G.K. Peptide-Ribose Assay		HbA1C formation	[35]
Epigallocatechin gallate	3T3-L1 preadipocytes	SIRT3	*CDKN1a* gene, IL-6	[36]
Phlorizin	ICR mice, PC12 cells	SOD, CAT, GPx, TAC, IL-2, ACh, Nrf2	MDA, IL-6, AST, ALT, AChE	[37]
Reynoutrin	CCD-986Sk fibroblast cells	type I procollagen	MMP-1	[10]
Anthocyanidin	3T3-L1 preadipocytes	NRF2, SIRT 3		[36]
Resveratrol	3T3-L1 preadipocytes	NRF2, SIRT 3		[36]
Isoquercitrin	CCD-986Sk fibroblast cells	type I procollagen, HAS2, TIMP-1, TGF-β	MMP-1, MMP-9	[34,38]
Rutin	In vitro assays: Hemoglobin-δ-Gluconolactone (δ-Glu) Assay, BSA-MGO Assay, BSA-Glucose Assay		HbA1C formation, methylglyoxal-medicated protein modification	[35]
Luteolin	In vitro assays: Hemoglobin-δ-Gluconolactone (δ-Glu) Assay, BSA-MGO Assay, BSA-Glucose Assay, G.K. Peptide-Ribose Assay		HbA1C formation, methylglyoxal-medicated protein modification, AGEs formation, subsequent cross-linking of proteins	[35]

ACh—Acetylcholine, AChE—Acetylcholinesterase, AGEs—advanced glycation endproducts, ALT—Alanine aminotransferase, AST—Aspartate aminotransferase, BAX—bcl-2-like protein 4, BCL-2—B-cell lymphoma 2, CAT—Catalase, CREB—cAMP response element-binding protein, COX-2—Cyclooxygenase-2, GPx—Glutathione peroxidase, HAS2—Hyaluronan synthase 2, HbA1C—Glycated hemoglobin, IL-2—Interleukin 2, IL-6—Interleukin 6, MMP-1—Matrix metalloproteinase-1, MMP-9—Matrix metalloproteinase-9, MSK1—Mitogen- and stress-activated protein kinase 1, NF-κB—Nuclear factor kappa B, NRF2—Nuclear factor erythroid 2-related factor 2, ROS—reactive oxygen species, RSK2—Ribosomal S6 kinase 2, SIRT 3—Sirtuin 3, SOD—Superoxide dismutase, TAC—Total antioxidant potential, TGF-β—Transforming bgrowth factor beta, TIMP-1—Metallopeptidase inhibitor 1.

### 2.2. Dietary Fiber, Vitamins, and Associated Nutrients

Apple pomace is exceedingly rich in dietary fiber, which not only adds nutritional value but also enhances the functional effects of polyphenols. On a dry-weight basis, pomace contains roughly 30–50% total fiber, including both insoluble components (cellulose, hemicellulose, lignin) and soluble pectins [16]. For instance, Rana et al. (2021) reported that approximately 42.6% total dietary fiber in pomace from a high-fiber apple cultivar (with 8% soluble pectin and 33% insoluble fiber approximately) [24]. These fibers have inherent health benefits. Insoluble fiber aids bowel motility and toxin elimination, while soluble pectin is fermentable, yielding short-chain fatty acids that reduce colonic inflammation [39]. Perhaps most intriguingly, pomace presents a polyphenol-rich fiber complex; phenolics can bind to fibers, which protects them from early digestion and promotes their delivery to the colon where they can act on the gut mucosa and microbiome [23,40]. This could help explain the observed prebiotic and anti-inflammatory effects of apple pomace in gut health studies. Moreover, fiber itself can sequester pro-oxidant metals and bile acids, indirectly contributing to an antioxidative, anti-aging physiological milieu. The dual presence of high fiber and polyphenols in pomace is therefore synergistic, a combination that slows carbohydrate release, improves metabolic profiles, and combats oxidative stress and inflammation, all relevant in aging and metabolic disease models [19].

In addition to fiber and polyphenols, apple pomace retains essential vitamins and micro constituents with antioxidant properties. Notably, substantial amounts of vitamin C and vitamin E persist in pomace. Ascorbic acid (vitamin C) has been reported at ~22–25 mg per 100 g dry pomace, making pomace a meaningful source of this water-soluble antioxidant (especially considering much of the apple’s vitamin C may leach into juice, the remaining pomace still contains biologically relevant levels). Tocopherols (vitamin E), largely concentrated in apple seeds, are also present—one analysis found approximately 5.5 mg vitamin E per 100 g pomace (dry basis) when seeds are included [10].

These vitamins work synergistically with polyphenols. A good example of this cooperation is ascorbate, which can regenerate oxidized phenols. Another one is vitamin E, which is a fat-soluble free radical scavenger that protects cell membranes from lipid peroxidation. Their presence is enhanced by the network of antioxidants in apple pomace. Pomace is also a source of minerals such as potassium, calcium, magnesium and iron [16], which support enzyme function and metabolic health. However, it should be added that minerals play a more indirect role in antioxidant protection (e.g., as cofactors of antioxidant enzymes). Finally, apple pomace contains triterpenoid compounds derived from apple peel, in particular ursolic acid. Ursolic acid has very beneficial effects. This acid is a pentacyclic triterpene with documented anti-inflammatory, antioxidant and even life-prolonging effects (e.g., improving muscle function and metabolism in older animals). Its presence in pomace (apple skins can contain >0.1% ursolic acid by weight) contributes to the bioactive profile [19]. Both the vitamins and phytochemicals present in pomace have high antioxidant potential. This, in turn, confirms the thesis that many micronutrients working together can holistically counteract the oxidative and inflammatory factors of aging.

Pomace’s lipid fraction, while small, deserves mention in the context of bioactive composition. Apple seeds (2–4% of pomace mass) contain oils rich in unsaturated fatty acids and tocopherols. The extracted apple seed oil is characterized by a high content of linoleic acid (≈45–60%) and oleic acid (≈20–30%), with a low proportion of saturates [41,42]. This gives the oil a heart-healthy fatty acid profile comparable to other nutritious seed oils. Though the absolute amount of oil in whole pomace is modest, its presence means pomace-derived ingredients carry small quantities of omega-6 linoleic acid and monounsaturated fats, which could support cell membrane integrity and normal physiology. Walia et al. (2014) demonstrated that apple seed oil from pomace has notable antioxidant capacity and even showed cytotoxic effects on cancer cell lines, hinting at bioactive compounds beyond just fatty acids (likely tocopherols and plant sterols) [42].

While the anti-aging significance of the pomace’s lipid fraction is less pronounced than that of polyphenols or vitamins, it nonetheless contributes to the overall nutrient synergy, for example, fats facilitate the absorption of fat-soluble phytonutrients like quercetin aglycones and carotenoids (trace amounts of carotenoids may also remain in pomace). Thus, even the minor lipid components play a supporting role in the health potential of apple pomace [43].

### 2.3. Variability by Cultivar and Processing Factors

It is crucial to recognize that the composition of apple pomace is not fixed; it varies with apple cultivar, growing conditions, and processing methods. These variations can create conceptual nuances when assessing pomace’s antioxidant potential and designing interventions for aging. Apple cultivar has a pronounced effect on pomace’s bioactive content. Generally, polyphenol levels are higher in more astringent or vividly colored apple varieties (which tend to have thicker peels and higher phenolic synthesis). For example, a comparative study of five cultivars in the Himalayan region showed that Royal Delicious apple pomace contained roughly double the total phenolic content (4.6 mg/g GAE) of other varieties and correspondingly higher antioxidant activity [24]. This variety’s pomace was especially rich in quercetin derivatives and phloridzin, as noted earlier, and also had the highest fiber content, suggesting a co-selection of traits for both polyphenols and fiber. By contrast, pomace from milder cultivars (e.g., Golden Delicious) showed lower phenolics (0.2 mg/g GAE) [24]. Persic et al. (2017) [44] in their study compared eight different apple varieties cultivated widely in Europe and locally in Slovenia. The results showed that Granny Smith and Topaz varieties had the highest total polyphenol content in pomace (0.35–0.53 mg/g GAE), while the native Majda variety had the lowest value of TPC (0.17 mg/g GAE) [44].

Such differences imply that the anti-aging efficacy of apple pomace could be optimized by cultivar choice; pomace from high-polyphenol apples may offer greater radical scavenging and bioactivity. There may also be structure–activity considerations across cultivars: for instance, red-skinned apples contribute anthocyanins (absent in green/yellow varieties) which add antioxidant and anti-inflammatory properties; some old or wild apple genotypes contain unusual phenolic profiles that could be potent but are less studied. These inter-cultivar differences invite further research—they hint that blending pomaces or selecting specific apple sources might enhance the consistency and potency of pomace-derived nutraceuticals.

Processing methods during and after juice extraction form another layer of variability. The way pomace is generated and handled can significantly influence its composition of bioactive compounds. For example, apples pressed for cider or cloudy juice (which may include more skin and seed breakage) can yield pomace with different polyphenol distributions than pomace from clear juice production. Likewise, drying and storage conditions of pomace are pivotal: gentle low-temperature drying or freeze-drying tends to better preserve heat-labile compounds like vitamin C and certain polyphenols, whereas high-temperature or prolonged drying can degrade these compounds [45,46].

Extraction studies also show that pretreatment affects yield; for example, more phenols were recovered from pomace fibers extracted in an aqueous methanol solution than from fibers extracted with acetone, highlighting that the solvent and method affect the measurable polyphenol content [47]. In addition, the enzymatic treatment used in some industrial extraction processes can either break the bonds between polyphenols and fibers (increasing the amount of free polyphenols) or cause phenol oxidation (if not controlled). Such nuances are important when planning the use of pomace: the natural antioxidant potential of fresh pomace may be higher than that of pomace that has undergone a lengthy industrial processing. However, a second important observation arises. It turns out that even processed pomace remains a “reliable source of pectin components” and antioxidants if it is properly processed [48].

In summary, standardization of pomace processing is crucial for the utilization of its bioactive anti-aging components. This observation leads to the conclusion that further research on optimized drying and extraction techniques is needed to valorize pomace [22].

### 2.4. Implications for Counteracting Aging Processes

The composite picture that emerges is that apple pomace contains a broad spectrum of bioactive compounds from polyphenols to fiber to vitamins that collectively can target multiple drivers of aging. The antioxidant capacity of pomace is not due to one “magic” ingredient but rather the ensemble of its constituents acting on different pathways. Polyphenols like quercetin, catechin, and phloridzin directly neutralize free radicals and reactive species, reducing oxidative damage to DNA, proteins, and lipids (a hallmark of aging) [16]. These same compounds also modulate cell signaling: for example, quercetin and phloridzin have been shown to activate Nrf2-driven antioxidant response elements and suppress pro-inflammatory NF-κB activation [25,27], thereby attenuating chronic inflammation often termed “inflammaging.” The dihydrochalcones further guard against metabolic aging by inhibiting glycation, as discussed, which can preserve protein function in tissues [28]. Meanwhile, the fiber in pomace improves gastrointestinal function and microbiome composition, which has systemic effects on immune regulation and even brain health as we age. Preclinical trials consistently demonstrate these benefits: apple pomace supplementation in animal models has improved antioxidant enzyme levels, lowered markers of inflammation, and enhanced metabolic parameters (like blood lipids and glucose) [49,50,51,52]. For instance, Skinner et al. (2018) [19] summarize that isolated apple pomace extracts improved lipid metabolism, antioxidant status, and gastrointestinal function in vivo. Such outcomes provide a biological rationale for pomace as an anti-aging functional ingredient.

Critically, the interactions among pomace’s constituents likely amplify its geroprotective impact. The co-presence of vitamin C can regenerate oxidized flavonoids, fiber can prolong polyphenol bioactivity along the gut and polyphenols in turn can protect other nutrients from oxidation. This network effect may resolve an apparent paradox: large, poorly absorbed polyphenols (like procyanidins) still contribute to health by acting in the gut lumen and being transformed into smaller phenolics that the body uses. Indeed, that paradox of high in vitro antioxidant activity vs. relatively low bioavailability, is addressed by considering the holistic role of pomace in the diet (working via gut microbes, local gut immunity, etc., beyond direct absorption) [23]. Smaller phenolic compounds (such as quercetin aglycone, catechin) may affect the function of multiple organs through their transport through the circulatory system, while larger compounds and fiber act in the gastrointestinal tract. Both groups of compounds are valuable in combating aging in various organ systems.

In summary, apple pomace has a rich and synergistic composition in terms of functionality. This is consistent with the main thesis that pomace can effectively counteract the aging process thanks to its antioxidant and bioactive content. Furthermore, the contents of compounds such as polyphenol complex, fiber, vitamins, unsaturated fatty acids, and triterpenes, pomace combats oxidative stress, inflammation, glycation, and intestinal dysbiosis are all the main factors associated with age-related degeneration. This evidence-based analysis confirms the hypothesis that apple pomace is not just agricultural waste, but a powerful collection of geroprotective agents. Such potential should not be underestimated.

In the remaining part of this article, we will focus on exploiting the above advantages of the chemical composition of pomace (taking into account its variability and bioavailability), which may pave the way for innovative nutritional strategies promoting healthy aging using what was once considered “apple waste” [16,19].

## 3. Extraction Methods for Bioactive Compounds

Efficient recovery of apple pomace polyphenols requires choosing extraction techniques that maximize yield and preserve antioxidant integrity, while remaining scalable and sustainable. A variety of methods from conventional solvent extraction to novel “green” technologies have been applied, each leveraging different mechanisms to liberate and solubilize bioactive compounds. These methods differ not only in extraction efficiency and selectivity, but also in their energy requirements, solvent use, and environmental footprint. A hypothesis-driven perspective is that the extraction method fundamentally influences which polyphenols are recovered and in what quantities, due to differences in mass-transfer driving forces and matrix disruption. Below, we compare conventional approaches with emerging green techniques, focusing on underlying biological processes and associated trade-offs (summarized in Table 2).

Despite methodological differences, all these extraction techniques aim to maximize the recovery of apple pomace’s antioxidant constituents while minimizing quality loss. Going forward, integrated approaches may yield the best results, for example, using an enzyme or PEF (pulsed electric field) pretreatment to break cell structures, followed by a short ultrasound or microwave extraction to rapidly solubilize phenolics. Such combinations could further boost yields and selectivity without proportional increases in energy or solvent usage [17].

Ultimately, the choice of extraction method in an industrial setting will depend on the desired product profile (broad-spectrum polyphenol powder vs. specific enriched fraction), scale and economic constraints, and environmental priorities. The growing body of research on apple pomace valorization consistently indicates that “green” extraction methods, especially those using renewable solvents (water or CO_2_) and energy-efficient physical processes, can produce high-quality, high-antioxidant extracts suitable for functional foods, cosmetics or animal nutrition, in alignment with sustainable production goals.

## 4. Mechanisms of Action Against Aging Processes

In this section, we will look at how the mechanisms that block aging at the cellular level work in practice. Apple pomace exhibits geroprotective effects through a number of interrelated mechanisms that affect the basic pathways of aging. The hypothesis linking these mechanisms assumes that the rich matrix of polyphenols and fiber modulates oxidative stress and inflammation while influencing cellular homeostasis processes such as autophagy, aging, and metabolism. The main modes of action of both selected apple polyphenols and apple pomace extracts are summarized in Table 1 and Table 3.

As a result, all these actions not only counteract molecular damage, but also dysregulation that causes aging. This is what makes apple pomace a multidirectional “cell aging inhibitor” [61].

One of the basic elements of the anti-aging effect of apple pomace is its strong weakening of reactive oxygen species (ROS) and protection of DNA integrity. Apple pomace contains large amounts of antioxidant polyphenols, such as quercetin glycosides, chlorogenic acids, and procyanidins. These directly scavenge free radicals [62].

Equally important, these phytochemicals activate endogenous antioxidant defense mechanisms via the Nrf2 pathway. In vivo studies have shown that apple polyphenols upregulate cytoprotective enzymes such as heme oxygenase-1 (HO-1) and NAD(P)H quinone oxidoreductase 1 (NQO1). At the same time, they downregulate the Nrf2 repressor protein 1 associated with Kelch-like ECH (Keap1) [63,64]. This change increases the ability of cells to respond to redox reactions and strengthens the integrity of tight junctions in tissues such as the intestines. This, in turn, indicates systemic antioxidant benefits [63].

By quenching ROS at the source and boosting antioxidant gene expression, apple pomace markedly reduces oxidative damage to biomolecules. Notably, apple peel extracts rich in quercetin and chlorogenic acid have been shown to protect DNA from oxidative insults in human cells [65]. Chen et al. (2025) report that apple polyphenols shield the genome from not only ROS-mediated lesions but also prevent from UV radiation and other genotoxic stressors [31,61].

In a human pilot trial, daily supplementation with polyphenol-rich apple pomace extract reduced markers of DNA oxidation (specifically oxidatively damaged purines) in patients, consistent with enhanced DNA repair or decreased damage [66]. By preserving genomic stability against oxidative and environmental injury, apple pomace components help break a key feed-forward cycle of aging [61].

A second major mechanism is the down-regulation of pro-inflammatory signaling pathways that drive “inflammaging.” Chronic, low-grade inflammation is a hallmark of aging, and apple pomace’s bioactive compounds show a remarkable capacity to quell this smoldering inflammation. In cellular and animal models, apple polyphenols consistently inhibit the NF-κB pathway, a pivotal regulator of inflammatory gene expression. Xu et al. (2015) found that apple polyphenol supplementation in ApoE^−/−^ mice suppressed the ROS/MAPK/NF-κB signaling axis, thereby lowering expression of adhesion molecules (VCAM-1, ICAM-1) and chemokines involved in vascular inflammation [67]. In this mechanism of action, the polyphenols prevented the degradation of the NF-κB inhibitor IκB-α and blocked p65 subunit translocation to the nucleus in oxidatively challenged endothelial cells in blood vessels. They also attenuated upstream MAPKs (p38, ERK1/2) that activate NF-κB, breaking the cycle of oxidative stress-induced inflammation [68]. Concurrently, inflammatory cytokine production (e.g., IL-6, TNF-α) is blunted. In aged rodent models, purified apple polysaccharides likewise inhibited NF-κB activation and downstream cytokines [69].

It can be noted that the positive anti-inflammatory effect occurs on several levels. By acting on both the NF-κB pathway and mitogen-activated protein kinase (MAPK), polyphenols from apple pomace change cell signaling from pro-inflammatory to more calm. There is also evidence that apple phenols affect the PI3K/Akt pathway, another signaling cascade that, when overactive, promotes inflammation and cell survival at the expense of longevity. Apple polyphenols can inhibit PI3K/Akt signaling, limiting an important factor that causes abnormal cell growth and inflammation [61,67]. This highly beneficial combination of anti-inflammatory actions indicates that apple pomace can effectively alleviate the chronic inflammatory burden associated with aging (known as inflammaging). In this way, it protects tissues from inflammatory damage and dysregulation.

In addition to its antioxidative and anti-inflammatory actions, apple pomace modulates essential cellular programs governing survival and turnover, including apoptosis, autophagy, and senescence processes that together shape tissue aging. At physiological levels, polyphenols in apple pomace display a dual role: they shield healthy cells from stress-induced apoptosis while selectively promoting the elimination of damaged or aberrant cells [32,68]. This duality helps remove potential senescent or pre-cancerous cells while preserving normal tissue function. For example, apple polyphenol treatments have been shown to modulate Bcl-2 family proteins, increasing pro-apoptotic factors (e.g., Bax, caspase-3) and lowering anti-apoptotic Bcl-2 in dysfunctional T-cells [69,70].

Such selective pro-apoptotic effects may aid in eliminating cells that would otherwise contribute to tissue aging and tumorigenesis. At the same time, apple pomace constituents bolster autophagy, the cell’s disposal and recycling system of unnecessary or damaged fragments. Enhancing autophagy is widely regarded as an anti-aging strategy because it clears damaged organelles (e.g., mitochondria) and protein aggregates that accumulate with age. Remarkably, apple polyphenols behave as caloric-restriction mimetics in this regard. Also they activate key autophagy regulators like enzymes SIRT1 and AMPK which enhanced cells autophagy and inhibit mTOR enzyme, a nutrient-sensing kinase that suppresses autophagy [71,72,73].

In aged high-fat-fed mice, Roy et al. (2022) showed that an apple polyphenol extract upregulated several autophagy-related proteins (ATG5, ULK1, Beclin-1) in the liver concomitant with increased SIRT1 expression [74]. These molecular changes translated into enhanced autophagic flux and reduced hepatic steatosis, highlighting the link between apple polyphenols, organelle quality control, and metabolic health in aging. Augmenting autophagy also has downstream effects on cellular senescence. By clearing damaged cellular components and reducing oxidative/inflammatory stress, apple pomace can delay the onset of the senescence program—a state in which cells irreversibly stop dividing and secrete inflammatory factors. Indeed, a recent study noted that apple-derived polysaccharides reduced the expression of aging and senescence-related proteins such as BDNF, PSD95 and SYP in the tissues of naturally aged mice [75].

This was accompanied by reduced apoptosis resistance and improved tissue function, indicating a broad rejuvenating effect at the cellular level. Although specific senescence markers were not detailed, the benefits are probably mediated by lowered levels of p16^INK4a^, p21^Cip1^ and SASP components, similarly to other plant polyphenols such as quercetin and fisetin. By preventing the onset of cellular senescence and enhancing the elimination of damaged cells via apoptosis and autophagy, apple pomace supports the maintenance of a functional cell pool, thereby helping to preserve tissue regenerative capacity and organ function with advancing age [36,76].

Apple pomace also promotes maintenance of the extracellular matrix, particularly in skin and connective tissues, which is crucial for counteracting age-related structural degeneration (wrinkling, loss of elasticity, etc.). The phenolic constituents from apple peels and pomace have demonstrated protective effects on collagen the most abundant structural protein that progressively breaks down during aging. In dermal fibroblasts, apple polyphenols significantly inhibit matrix metalloproteinase-1 (MMP-1), the collagenase responsible for cleaving type I collagen fibers. Park et al. (2014) isolated several flavonoids from unripe apples and showed that compounds like quercetin glycosides potently suppressed MMP-1 activity (IC_50_ in low micromolar range) [34].

Simultaneously, those polyphenols boosted the synthesis of new collagen, as evidenced by a ~78% increase in type I procollagen production in treated fibroblasts. By reducing collagen degradation and increasing collagen synthesis, apple pomace components can help maintain the dermal matrix density that normally declines with age [34].

Complementing these in vitro findings, Lee et al. (2021) reported that isoquercitrin a major apple peel polyphenol protected skin cells from UV-induced photoaging by modulating the MMP/TIMP (tissue inhibitor of metalloproteinases) balance [38]. Isoquercitrin treatment of UVB-exposed human fibroblasts markedly downregulated MMP-1 and MMP-9 while upregulating TIMP-1 and collagen I gene expression. This effectively prevented collagen breakdown and even increased net collagen content under photostress conditions.

As a result of the above reactions, we obtain the desired effect. The end result is the preservation of the structure and function of the skin. Several significant benefits of this effect have been described in human studies. Let us take a closer look at the details.

Shoji et al. (2020) showed that 12 weeks of oral apple polyphenol supplementation significantly protected skin from UV aging [77]. Women taking apple polyphenol tablets had less UV-induced erythema and melanin pigmentation compared to placebo, indicating resistance to photoaging outcomes. Although skin hydration parameters were unchanged, the improvement in UV tolerance suggests apple pomace polyphenols strengthened the skin’s defense against solar aging [77]. Taken together, by safeguarding collagen from enzymatic destruction, enhancing collagen synthesis, and mitigating UV damage, apple pomace mayhap they could slow down the dermal aging process, maintaining skin elasticity and reducing wrinkle formation.

**Table 3:** Effect of Apple Pomace Extracts in vivo and in vitro.

Research Model	Upregulation	Downregulation	References
Pig and IPEC-J2 cell models	intestinal morphology, production of secretory immunoglobulin A, TAC, Nrf2/Keap1	Population of *Escherichia coli*	[63]
ApoE^−/−^ mice	HDL, GPx, CAT, SOD, PPARα	atherosclerotic lesions, hepatic steatosis, LDL, TG, CCL-2, VCAM-1, SCAP, macrophage infiltration in the aortic root plaque, inflammatory cells infiltrations, ox-LDL-induced endothelial dysfunction, monocyte adhesion to RAECs, ROS/MAPK/NF-κB	[67]
Male Swiss albino mice	SOD, GSH, LPO, Nrf2	ALT, AST, ALP, necrotic changes	[68]
Male C57BL/6 mice	serum albumin/globulin ratio, hepatic steatosis, LKB1, phosphorylated-AMPK, phosphorylated-ACC, SIRT1, *Cyp27a1* gene, HSL, ATG5, Ulk1, Becn1, Akkermansia probiotics abundance	TG, TC, mTOR, p70 s6k, HMGCR, Srebp-1c, Fas receptor, FOXO1, ratio of Firmicutes/Bacteroidetes	[71,74]
Kunming mice	miR-22-3p/SIRT 1	oxidative damage, IL-1β, IL-6, TNF-α, Iba1, caspase 3, caspase 9, BAX	[72]
Male Obese Zucker rats (OZR)	insulin sensitivity, GLUT4 translocation, GIR, PI3K, PPARγ		[78]

ACC—Acetyl-CoA carboxylase, ALP—Alkaline phosphatase, ALT—Alanine aminotransferase, AMPK—5′ adenosine monophosphate-activated protein kinase, AST—Aspartate aminotransferase, ATG5—Autophagy protein 5, BAX—bcl-2-like protein 4, Becn1—Beclin-1, CAT—Catalase, CCL-2—chemokine (C-C motif) ligand 2, FOXO1—Forkhead box protein O1, GIR—Glucose infusion rate, GLUT4—Glucose transporter type 4, GPx—Glutathione peroxidase, GSH—Glutathione, HDL—High-Density Lipoprotein, HMGCR—3-hydroxy-3-methyl-glutaryl-coenzyme A reductase, HSL—Hormone-sensitive lipase, Iba1—Ionized calcium-binding adapter molecule 1, IL-1β—Interleukin 1 beta, IL-6—Interleukin-6, Keap1—Kelch-like ECH-associated protein 1, LKB1—Liver kinase B1, LDL—Low-density lipoprotein, LPO—Lipid peroxidation, MAPK—Mitogen-activated protein kinase, mTOR—Mammalian target of rapamycin, NF-κB—Nuclear factor kappa B, Nrf2—Nuclear factor erythroid 2-related factor 2, ox-LDL—Oxidized low-density lipoprotein, p70 s6k—Ribosomal protein S6 kinase beta-1, PI3K—Phosphoinositide 3-kinase, PPARα—Peroxisome proliferator-activated receptor α, PPARγ—Peroxisome proliferator-activated receptor γ, RAECs—rat aortic endothelial cells, ROS—reactive oxygen species, SCAP—SREBP cleavage-activating protein, SIRT 1—Sirtuin 1, SOD—Superoxide dismutase, Srebp-1c—Sterol regulatory element-binding protein 1, TAC—Total antioxidant potential, TC—Total cholesterol, TG—Triglyceride, TNF-α—Tumor necrosis factor, Ulk1—Serine/threonine-protein kinase, VCAM-1—Vascular cell adhesion molecule-1.

Moving on to the next aspects, apple pomace favorably modulates metabolic markers and pathways that are tightly linked to aging, including lipid metabolism, glucose homeostasis, and protein glycation. The dietary fiber in apple pomace (rich in pectin and hemicelluloses) and its polyphenols act synergistically to improve metabolic health. High-fiber apple pomace can sequester bile acids and cholesterol in the gut, thereby reducing lipid absorption and improving blood lipid profiles. Animal studies have documented that adding apple pomace to the diet lowers total and LDL-cholesterol levels without adverse effects. For instance, Ravn-Haren et al. (2018) found that apple pomace supplementation in rats significantly decreased total LDL and IDL (intermediary-density lipoprotein) cholesterol relative to controls, an effect partly attributed to increased production of short-chain fatty acids (SCFAs) from fiber fermentation and greater fecal bile acid excretion [30].

Another study noted that pomace diets reduced hepatic cholesterol accumulation by ~11%, which underlines the lipid-lowering potential of pomace fiber.

Such improvements in cholesterol metabolism can mitigate atherosclerosis and cardiovascular aging. In parallel, apple polyphenols directly influence glucose metabolism and insulin sensitivity. Manzano et al. (2016) showed that an apple polyphenol extract enhanced insulin sensitivity in insulin-resistant rats, increasing the glucose infusion rate by 45% [78]. In this case, the polyphenols acted on skeletal muscle cells to promote GLUT4 glucose transporter translocation, thus upping glucose uptake, in part via activation of the PI3K/Akt pathway and PPARγ signaling.

Let us look closely the insulin-mimetic characteristic of apple pomace. This insulin-mimetic or insulin-sensitizing effect aligns with epidemiological data that high apple/polyphenol intake correlates with better glycemic control and lower risk of type 2 diabetes. By blunting post-prandial hyperglycemia, apple pomace may also reduce formation of advanced glycation end-products (AGEs), which are sugar-derived protein crosslinks that accumulate with age and damage tissues. Polyphenols in general can inhibit AGE formation by trapping reactive carbonyl intermediates and through antioxidant activity [79]. Apple’s polyphenols (e.g., quercetin, phloridzin) have been noted to scavenge methylglyoxal a potent glycating agent thereby slowing protein glycation. Additionally, apple pomace’s ability to lower systemic oxidative stress and inflammation curtails the oxidative glycation pathways that exacerbate AGE generation [35].

This has particular relevance for collagen in skin, as AGEs contribute to stiffness and loss of elasticity. By reducing AGEs, apple pomace indirectly supports tissue rejuvenation. A similar situation applies to the collagen that builds arteries. Another metabolic dimension of apple pomace is its interaction with the gut microbiome. The non-digestible fibers and polyphenols in pomace serve as substrates for gut bacteria, selectively promoting beneficial genera (like *Lactobacillus* and *Bifidobacterium*) while suppressing harmful ones (like *Helicobacter* and *Bilophila*) [63].

Fermentation of apple pomace yields SCFAs (acetate, propionate, butyrate) that have systemic anti-inflammatory and metabolic effects for example, improving insulin signaling and providing energy for intestine cells. Yin et al. (2023) observed that apple polysaccharides in aged mice shifted the microbiota and restored gut barrier integrity, resulting in reduced circulating endotoxin and inflammatory load [75]. Thus, some anti-aging benefits of apple pomace may originate in the gut (locally reducing inflammation and entry of endotoxins) and ripple outward to systemic metabolism. This gut-centric mode of action complements the direct antioxidant and signaling effects of apple pomace compounds in tissues.

### Conceptual Nuances and Limitations

While the multi-pathway benefits of apple pomace are compelling, it is important to consider the bioavailability and context of its bioactive constituents. Many polyphenols in apple pomace, such as phloridzin, are glycosides that are poorly absorbed in the small intestine and mainly metabolized by colonic microbes. In the human trial by Grindel et al. (2014) only trace plasma levels of phloridzin (~12.7 ng/mL) and its aglycone phloretin (~19.3 ng/mL) were detected after weeks of supplementation [66].

Such low systemic levels suggest that local actions in the gut and the activity of polyphenol metabolites (produced by microbiota) are key contributors to the observed anti-aging effects, rather than high circulating concentrations of intact polyphenols. In other words, apple pomace may act as a synbiotic delivering fermentable fiber and polyphenols that together nurture a healthier microbiome and produce metabolites (e.g., urolithins from ellagitannins, or phenyl-γ-valerolactones from procyanidins) with systemic antioxidant and anti-inflammatory properties. This means the efficacy of apple pomace could vary with an individual’s gut flora composition. Furthermore, while in vitro and animal studies show clear benefits of understanding the mechanisms, translating these findings to human aging will require long-term clinical data. Dosage is another consideration: the amounts of polyphenols used in some experiments may be difficult to attain through diet alone, though concentration by extraction (as in supplements) can overcome this. Finally, the balance between pro-oxidant and antioxidant effects of polyphenols at high doses should be acknowledged. At elevated concentrations these compounds can induce mild oxidative stress that activates protective cellular pathways, such as Nrf2 signaling. This dose-dependent duality needs to be carefully considered in the context of long-term use [80]. Despite these nuances, the preponderance of evidence supports a net beneficial role for apple pomace against aging processes.

**Figure 1:**
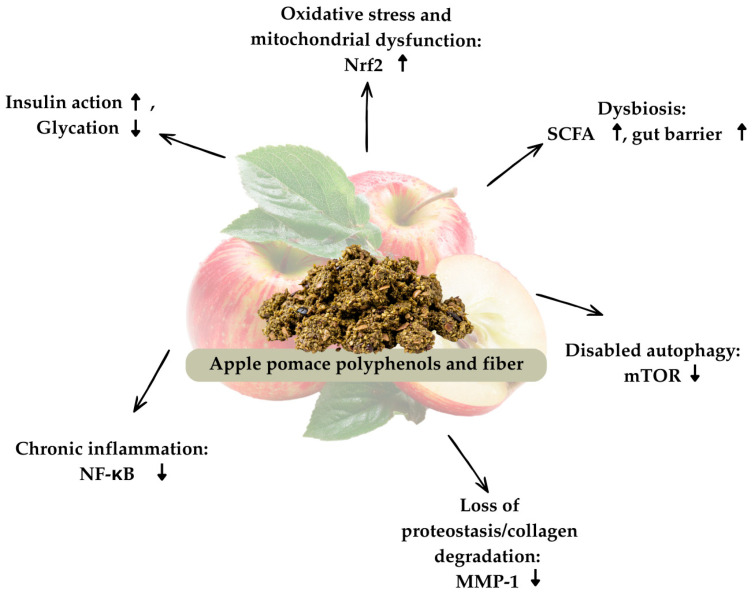
Overview of apple pomace bioactive compounds in relation to hallmarks of aging.

In summary, apple pomace, once a mere agricultural waste, emerges as a functional food ingredient with multifaceted anti-aging potential. Its polyphenols and fibers orchestrate a network of protective mechanisms: quenching ROS and guarding DNA, suppressing inflammatory cascades (NF-κB, MAPK, PI3K/Akt), modulating apoptosis and autophagy to rejuvenate cells, preserving extracellular matrix and collagen, and optimizing metabolic health by lowering lipids, improving insulin action, and reducing glycation as summarized in Figure 1. These diverse mechanisms converge to counteract the physiological drivers of aging and age-related disease, aligning with the thesis that apple pomace’s rich antioxidant profile can promote healthy aging across multiple organ systems. By intervening at several biological levels—from the genome to the microbiome apple pomace exemplifies a holistic approach to mitigating aging processes. This broad spectrum of action means that apple pomace can be used not only as a health-improving nutraceutical but also as an anti-aging agent [61].

Although preclinical evidence from in vitro and rodent studies is strong, several limitations should be noted. First, many polyphenols in apple pomace, especially polymeric procyanidins, have low systemic bioavailability and exert their effects primarily locally in the gut through microbial metabolites. Second, most experiments employ high doses that are difficult to reach with normal dietary intake. Third, polyphenols can be beneficial at low or moderate doses but may become pro-oxidant at very high concentrations, highlighting the need for thorough dose–response studies. Finally, long-term randomized controlled trials in elderly humans are urgently required to confirm these findings and support clinical recommendations.

## 5. Applications of Apple Pomace

We have already discussed the health benefits of pomace. However, we have not yet mentioned another extremely important method of use, namely in energy, food production and cosmetics. The growing annual production of apple pomace is forcing scientists to look for solutions for its use in various industries.

One such sector is the renewable energy sector, where pomace is used to produce biofuel, bioethanol, biogas, and biochar [81]. We are also seeing the use of pomace in agriculture and animal feed [82]. A growing number of studies are using apple pomace as an ingredient in food production and as a potential cosmetic ingredient [7,16,22,83].

### 5.1. Usage of Apple Pomace as Ingredients in Food

Table 4 presents an overview of research on the use of apple pomace in food production. These studies can be divided into three groups: bakery products, meat products, and dairy products. In the first group, apple pomace flour is used in the production of bread, cookies, muffins, biscuits, cereal crispbread, snacks, etc. [84,85,86]. The common denominator among these studies is that the addition of apple pomace results in an improvement in the nutritional and antioxidant properties of the products. Attention is drawn to increased dietary fiber and polyphenol content. However, the studies also note altered texture, color, and sensory perception [87,88,89]. In meat products, apple pomace was used as a substitute for fillers such as starch or breadcrumbs. The sensory experience, consistency, and firmness of the product depend on the type of meat with which the apple pomace was mixed. However, meat products containing apple pomace are characterized by superior nutritional and antioxidant value [84,90,91]. The positive effect of adding apple pomace on the structure and composition was observed in dairy products such as yogurt and cheese [92,93,94].

### 5.2. Potential Application of Apple Pomace in Cosmetics Industry

Recent studies highlight the strong potential of apple-derived compounds for anti-aging and anti-wrinkle cosmetics. Ursolic acid from Annurca apples shows potent elastase inhibition and, together with polyphenols like quercetin, reduces wrinkles and improves skin hydration, proving safe for topical use [99]. Phenolic extracts from Arisoo and ‘Summer King’ apples also exhibit significant anti-wrinkle activity, with ethanol extracts generally providing higher phenolic content and bioactivity than water extracts [7,100].

Apple-derived nanovesicles (ADNVs) act by downregulating pro-inflammatory pathways (NF-κB, TLR4), promoting collagen synthesis, reducing metalloproteinase activity, and limiting extracellular matrix degradation, making them effective in gels and patches for skincare [101]. Cosmetic formulations combining wild apple extracts, alpha-hydroxy acids (AHAs), and polyphenols demonstrate stable antioxidant activity, improve skin hydration, reduce hyperpigmentation, and are non-irritating in vivo [102]. Similarly, creams with *Malus* sp. extracts and rutin decrease apoptotic cell formation and UV-induced damage, confirming their protective and anti-aging properties [103]. Polyphenol-rich extracts from Aomori Hiba, wild apples, and Unripe Apple Extract (UAE) demonstrated antioxidant, anti-inflammatory, and collagen-promoting effects, enhancing skin protection against UVB-induced oxidative stress, DNA damage, and inflammation [7,104].

Apple extracts including hydroalcoholic formulations, stem cell extracts (ASCs), and oleolites show efficacy in reducing pro-inflammatory markers (e.g., TNF-α, IL-1β), regulating collagen synthesis, decreasing metalloproteinase activity, and preventing extracellular matrix degradation. These bioactive compounds also improve skin hydration, reduce hyperpigmentation, and mitigate wrinkle formation [99,105].

Recent studies on Formosan apples have identified key bioactive compounds, particularly 3-hydroxyphloretin and catechol, which exhibit strong antioxidant activity and effectively reduce tyrosinase activity in human epidermal melanocytes, suggesting potential use as skin-lightening agents in cosmetics [106]. Clinical and in vitro research on apple polyphenols, including procyanidins, demonstrated their ability to inhibit melanin synthesis, protect against UV-induced oxidative damage, and support skin health by modulating gut microbiota and reducing inflammation. These findings highlight apple-derived compounds as promising natural ingredients for preventing pigmentation, maintaining skin lightness, and protecting against UV-related skin damage [77].

## 6. Future Perspectives

Research conducted on the use of apple pomace shows its considerable potential. Studies on the production of food products with apple pomace additives show that they improve nutritional properties and provide a range of antioxidants. Improving the sensory and physical properties of products made with apple pomace remains a challenge, especially in bakery products [83]. In the longer term, we should see the gradual emergence of foods containing apple pomace as a health-promoting alternative to conventional products.

Diverse methods for extracting compounds from apple pomace open up possibilities for their use in the production of cosmetics, nutraceuticals, and dietary supplements. The development of research on the use of apple pomace extracts in cosmetics shows that this is a newly emerging trend, and further research will be conducted on this topic.

However, one of the key issues in research on the use of apple pomace and its derivatives should be their safety and toxicity. The rationale for this should primarily be the plant protection products (e.g., pesticides, fungicides) used in apple cultivation. The use of increasingly new generations of plant protection products necessitates the development of new research methods to effectively determine these compounds and prevent their excessive accumulation in apples and subsequent processed products, including apple pomace.

## 7. Summary and Conclusions

In summary, apple pomace, the fibrous byproduct of apple juice and cider processing has emerged from an agricultural waste into a multifunctional resource with significant antioxidant and anti-aging potential. It is rich in polyphenols (e.g., flavonoids, phenolic acids), dietary fiber (notably pectin and insoluble fractions), and essential nutrients such as vitamins, offering a matrix of bioactive compounds that work synergistically to counteract aging processes.

Its key mechanisms of action include neutralizing reactive oxygen species and enhancing cellular antioxidant defenses, suppressing chronic inflammation (mitigating “inflammaging”), supporting a healthy gut microbiome and metabolic homeostasis, and preserving collagen as well as overall tissue integrity. Through these multi-targeted effects, apple pomace addresses several fundamental hallmarks of aging and helps protect against age-related degeneration. Although strong preclinical evidence demonstrates the multi-target antioxidant and anti-aging potential of apple pomace through modulation of Nrf2, NF-κB and SIRT1 pathways, clinical validation remains limited.

These insights are increasingly being translated into practice. Apple pomace is now being upcycled into functional food products (e.g., polyphenol- and fiber-enriched baked goods and fermented dairy foods), used in cosmeceutical formulations (anti-wrinkle creams and serums), and developed as nutraceutical supplements to support healthy aging. Such applications not only leverage the synergies of working apple pomace (antioxidant, anti-inflammatory, and prebiotic effects working in concert) but also align with sustainability and circular economy principles by converting waste into value-added products.

As an economical and naturally sourced product with a favorable safety profile, apple pomace is a promising addition to a range of strategies promoting longevity and healthy aging. There is no doubt that further research is needed in the future to fully exploit its potential. Such research must include long-term clinical trials, optimization of extraction and delivery of bioactive substances, and standardization of product formulations.

To maximize the environmental benefits of this research, it must be conducted in accordance with the principles of the One Health and 5R strategies. Regulatory clarity will also be essential to ensure consumer safety. In this regard, it is also important to establish clear boundaries between food health claims and medical applications.

In conclusion, apple pomace is a good example of how an evidence-based, recycled ingredient can contribute to healthy aging by combining nutrition science with sustainable innovation. Future research should prioritize long-term human trials and standardization of extracts in accordance with the principles of the circular economy and One Health approach.

## Figures and Tables

**Table 2:** Comparison of extraction methods for recovering polyphenols from apple pomace, including typical yields and process considerations.

Extraction Method	Polyphenol Yield and Efficiency	Energy Use (Process Intensity)	Solvent Usage	Environmental Footprint and Notes
Conventional solvent (maceration)	~15–22 mg GAE/g (dry basis) with optimized 50–80% ethanol [53]. Broad spectrum extraction; prolonged contact can reach high yields but may extract undesired solids.	High—long extraction times (1–24 h) often with heating/stirring.	High—large volumes of organic solvent (ethanol or methanol) required; solvent must be evaporated off (energy-intensive).	Significant solvent waste and emissions unless solvents are recycled [54]. Higher risk of polyphenol oxidation over long times. Well-known, simple, but not environmentally benign.
Soxhlet (continuous hot solvent)	Typically yields comparable to exhaustive maceration (nearly complete recovery of extractables). Operates at solvent’s boiling point, which can degrade heat-sensitive phenolics [54].	Very high—solvent is kept boiling for many hours (thermal energy input continuous).	Very high—uses solvent in a loop; total solvent volume is large (though recycled in apparatus).	Efficient extraction but poor sustainability. Solvent recycling partly mitigates chemical waste, yet overall energy demand and potential compound degradation make it less “green.” Not easily scalable to bulk (mainly lab-scale).
Ultrasound-assisted (UAE)	Fast and efficient: >90% of total phenolics extracted in ~10 min [54]. Yields ~10–13 mg GAE/g with water alone (higher with ethanol) in short bursts. Cavitation disrupts cells, enhancing mass transfer [54].	Moderate—ultrasonic transducers require power, but short duration. Some additional heating of solvent (often 40–60 °C) to aid extraction.	Moderate—solvent volume can be lower than in maceration; water or ethanol–water commonly used. No need for exotic solvents.	Relatively low footprint due to reduced solvent and time. Lower CO_2_ emissions than long conventional extractions. However, if scaled, energy efficiency depends on ultrasonic equipment (UAE at 60 °C with high solvent ratios can increase footprint) [54]. Generally considered a green method with minimal chemical waste.
Microwave-assisted (MAE)	Very high efficiency: similar or slightly higher yields than UAE and conventional in a few minutes [54], e.g., ~13 mg GAE/g in 5 min with water [54]. Effective cell rupture via rapid internal heating [54,55].	Low–Moderate—microwaves heat only the sample/solvent directly; energy input is brief but intense (e.g., 300 W for 5 min). Overall energy per extraction is low.	Low—works well with small solvent volumes (10:1 to 20:1 *v*/*w*); water or benign solvents can be used. No prolonged solvent reflux needed.	Very favorable environmental profile: short processing translates to low energy per yield. In LCA, MAE showed the lowest climate impact among methods [54]. Virtually no solvent emissions if water is used. Caution: requires electrical infrastructure; scale-up needs multiple microwave units (which could increase energy if not optimized).
Enzymatic (e.g., pectinase-assisted)	Improves yield significantly (often +15–30% TPC vs. no enzyme) [17] by liberating bound polyphenols. Absolute yields vary (depending on enzyme and pomace); often followed by a quick solvent or water rinse to collect released phenolics.	Low—operates at mild temperature for 1–6 h; energy mainly for maintaining 40–50 °C and agitation.	Very low—uses water as reaction medium; no organic solvent required until perhaps a final extraction of the liquid (which can sometimes be avoided). Enzyme protein is the main added “reagent.”	Environmentally friendly and selective. No toxic solvents; minimal energy. Enzymes are biodegradable, but their production has some footprint. Overall greatly reduces chemical waste. Not instant—slower throughput. Cost can be higher due to enzyme inputs. Scalable in standard bioreactors.
Supercritical CO_2_ (SFE)	Can selectively extract ~50–80% of pomace polyphenols (especially less-polar ones). Yields of key antioxidants can equal or exceed solvent extraction under optimized high-pressure conditions [56,57], though total mass extracted may be lower [57,58]. Often produces smaller, more potent extract fractions.	High—requires pressurization to 10–30 MPa and temperature control (35–70 °C). Continuous flow systems consume significant energy, but CO_2_ recycle and heat recovery ameliorate this.	Minimal—CO_2_ is the main solvent (recyclable); a food-grade co-solvent (ethanol) is often used at ~5–15% to help dissolve polar phenolics [57] (this co-solvent is much less than in CSE).	Very green solvent usage (essentially no organic solvent waste; CO_2_ is reused). Carbon footprint depends on energy source for compression—if renewable energy is used, SFE’s environmental impact is low. No residual solvent in extracts. High equipment and operating cost; best suited for high-value nutraceutical extracts.
Pressurized liquid (PLE) (Subcritical water, Accelerated Solvent Extraction)	Highly efficient—achieves comparable TPC yields to conventional extraction in a fraction of the time [57,59]. For instance, water at 120–150 °C (subcritical) can extract a wide range of polyphenols; total yields ~10–18 mg GAE/g have been reported depending on conditions [60]. Tuning solvent polarity with temperature allows broad-spectrum extraction.	Moderate—energy needed to heat solvent to 100–200 °C and maintain pressure (~5–15 MPa), but extraction is rapid (minutes). Batch or flow-through systems can recover heat between cycles.	Low–Moderate—uses water or benign solvents under pressure. Solvent-to-solid ratios are lower than in ambient extraction because high diffusivity yields more from less solvent. Often <20:1 *v*/*w* and solvent can be recycled.	Green profile, especially with water as solvent: eliminates organic solvents and cuts extraction time (reducing overall energy). Some thermal degradation possible at very high T, but no toxic by-products. Equipment needs pressurization capability. Overall footprint is favorable; one study noted replacing organic solvent with water in PLE significantly reduced environmental impact of polyphenol recovery [54].

**Table 4:** Applications of apple pomace as ingredients in foods.

Research	Product	Conclusions
Jung et al. (2015) [84]	Cookies, Muffins, Chicken patties, Beef jerky	The partial replacement of wheat flour with apple pomace flour in cookies and muffins did not negatively affect their physicochemical or textural qualities compared to the control samples. When wet apple pomace was incorporated directly into meat products, it lowered their firmness but increased the levels of dietary fiber, pectin, and antioxidant activity.
Rocha Parra et al. (2015) [95]	Gluten-free bread	A product with acceptable characteristics was achieved; however, the dynamic moduli of the batters, the specific volume, and the crumb texture varied depending on the proportion of apple pomace and water used. Increased fiber content led to reduced crumb cohesion and elasticity, as well as a decrease in specific volume.
Bchir et al. (2014) [96]	Bread	The resulting product had increased fiber content and properties similar to white bread. However, the bread enriched with apple pomace had a comparatively different color.
Valková et al. (2022) [86]	Bread	The findings indicate that incorporating 10% apple pomace powder into bread recipes can be a promising way to produce baked goods with enhanced nutritional value while maintaining desirable quality and sensory characteristics.
Kruczek et al. (2023) [89]	Cookies	An increasing proportion of apple pomace led to a notable rise in total, soluble, and insoluble fiber content, but also made the cookies harder, darker in color, and smaller in volume.
Mir et al. (2017) [85]	Gluten-free brown rice crackers	Brown rice crackers with 9% apple pomace were well accepted and showed higher antioxidant activity, polyphenols, flavonoids, dietary fiber (especially insoluble), and minerals, while also affecting color and texture. This confirms apple pomace as a valuable functional ingredient for bakery products.
Konrade et al. (2017) [97]	Cereal crispbread	The addition of pomace resulted in an increase in total dietary fiber compared to the control group. Products with pomace addition exhibited a firmer consistency.
Alongi et al. (2019) [87]	Biscuits	The addition of apple pomace resulted in cookies with a lower glycemic index compared to the control group. However, the addition of pomace affected the product’s sensory and physical properties.
Drożdż et al. (2014) [88]	Extruded snacks	Increase in total phenolic content and antioxidant activity has been observed after adding of apple pomace to the snacks. Change of color has been observed in comparison to the control product
Kapoor et al. (2023) [98]	Jam	The study showed that jams made with apple pomace are rich in phenolic compounds, carotenoids, dietary fiber, and possess strong antioxidant activity.
Yadav et al. (2016) [90]	Chicken sausage, Chicken nuggets	The addition of apple pomace enhanced the antioxidant activity and color of the sausages while simultaneously increasing the product’s firmness. In the case of the nuggets, a product with satisfactory sensory properties was obtained.
Younis and Ahmad (2018) [91]	Buffalo beef patties	Adding apple pomace powder to buffalo meat patties improved cooking yield, emulsion stability, water retention, as well as patty diameter and thickness. Compared to the control, the fortified patties showed greater firmness, toughness, hardness, and cohesiveness, supported by a more uniform and stable structure.
Wang et al. (2020) [94]	Yogurt and yogurt drink	The study showed that apple pomace modified the structure of stirred yogurt, enhancing firmness, cohesiveness, and viscosity while significantly decreasing whey separation during cold storage. When added to a diluted yogurt system, apple pomace helped stabilize the acidic drink and reduced protein sedimentation. Incorporating apple pomace into already fermented milk gels allowed concentrations up to 6% (*w*/*w*), turning stirred yogurt and yogurt beverages into effective carriers of dietary fiber and phytochemicals.
Issar et al. (2017) [92]	Acidophilic yogurt	Based on sensory evaluation, yogurt with 5% apple fiber was rated the highest and was therefore selected as the optimal formulation for producing fiber-enriched acidophilus yogurt with favorable quality and sensory characteristics.
Mileriene et al. (2023) [93]	Cheese	Adding apple pomace to cheese influenced its composition (moisture, protein, fat, carbohydrates, and fiber), texture, color (lightness, redness, and yellowness), and overall sensory acceptability. The supplementation slightly increased L. lactis LL16 counts by day seven, indicating a beneficial effect of apple pomace on bacterial survival. A symbiotic interaction between apple pomace and LL16 was observed in proteolysis on day one, which may have contributed to improved sensory quality.

## Data Availability

No new data were created or analyzed in this study. Data sharing is not applicable to this article.
